# Supplementary material for: Novel Driver Strength Index highlights important cancer genes in TCGA PanCanAtlas patients
Source: PeerJ. 2022 Aug 11;10:e13860. doi: 10.7717/peerj.13860 (PMC9375969; doi:10.7717/peerj.13860)
Supplement: Supplemental Information 2 [file peerj-10-13860-s002.zip › COHORTS/genes plots/age/2021_8_16_14_9_distribution_cohorts_males_85_.pdf]

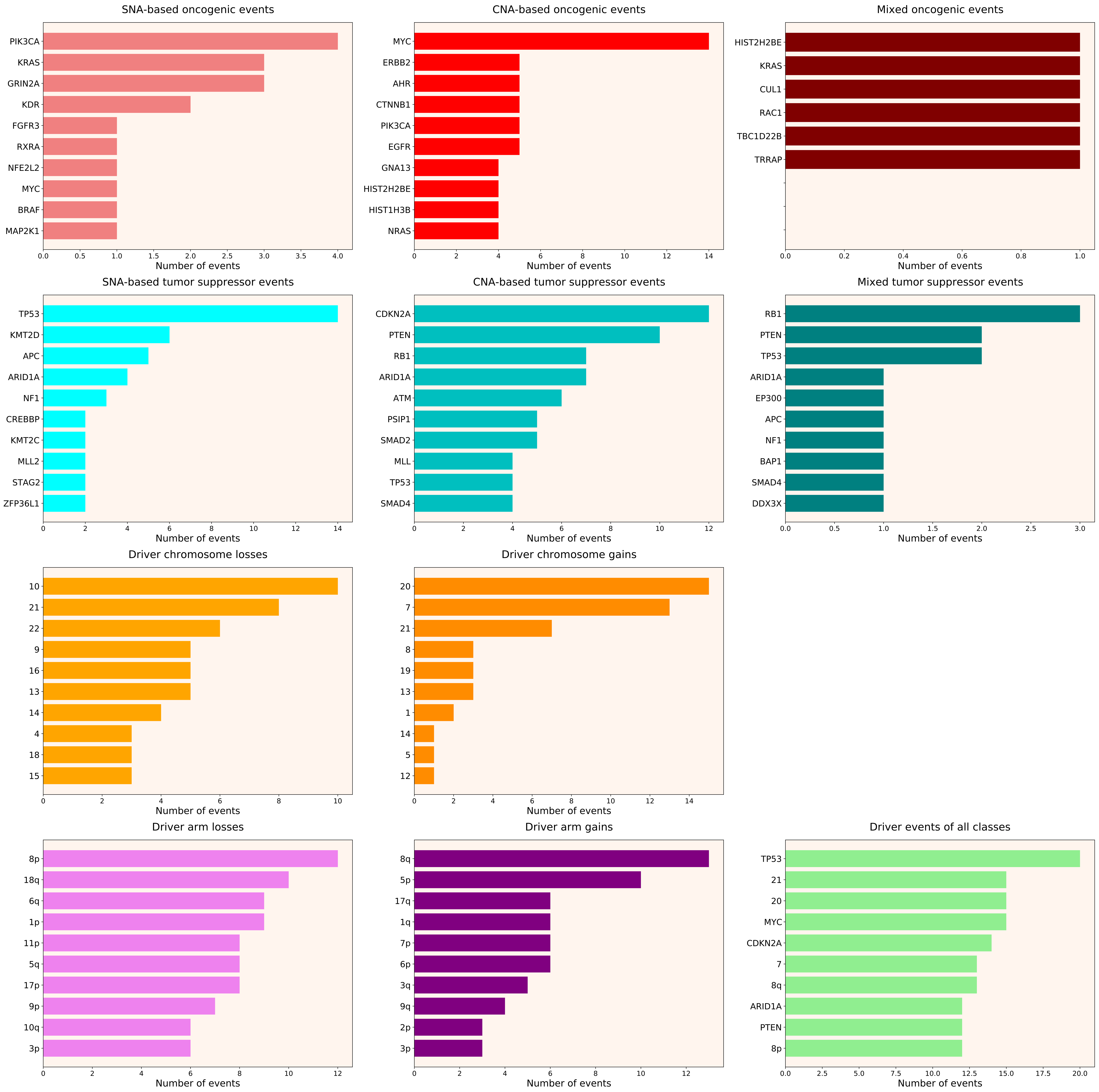
SNA-based oncogenic events
PIK3CA
KRAS
GRIN2A
KDR
FGFR3
RXRA
NFE2L2
MYC
BRAF
MAP2K1
0.0 0.5 1.0 1.5 2.0 2.5 3.0 3.5 4.0
Number of events
CNA-based oncogenic events
MYC
ERBB2
AHR
CTNNB1
PIK3CA
EGFR
GNA13
HIST2H2BE
HIST1H3B
NRAS
0 2 4 6 8 10 12 14
Number of events
Mixed oncogenic events
HIST2H2BE
KRAS
CUL1
RAC1
TBC1D22B
TRRAP
0.0 0.2 0.4 0.6 0.8 1.0
Number of events
SNA-based tumor suppressor events
TP53
KMT2D
APC
ARID1A
NF1
CREBBP
KMT2C
MLL2
STAG2
ZFP36L1
0 2 4 6 8 10 12 14
Number of events
CNA-based tumor suppressor events
CDKN2A
PTEN
RB1
ARID1A
ATM
PSIP1
SMAD2
MLL
TP53
SMAD4
0 2 4 6 8 10 12
Number of events
Mixed tumor suppressor events
RB1
PTEN
TP53
ARID1A
EP300
APC
NF1
BAP1
SMAD4
DDX3X
0.0 0.5 1.0 1.5 2.0 2.5 3.0
Number of events
Driver chromosome losses
10
21
22
9
16
13
14
4
18
15
0 2 4 6 8 10
Number of events
Driver chromosome gains
20
7
21
8
19
13
1
14
5
12
0 2 4 6 8 10 12 14
Number of events
Driver arm losses
8p
18q
6q
1p
11p
5q
17p
9p
10q
3p
0 2 4 6 8 10 12
Number of events
Driver arm gains
8q
5p
17q
1q
7p
6p
3q
9q
2p
3p
0 2 4 6 8 10 12
Number of events
Driver events of all classes
TP53
21
20
MYC
CDKN2A
7
8q
ARID1A
PTEN
8p
0.0 2.5 5.0 7.5 10.0 12.5 15.0 17.5 20.0
Number of events
